# Response to the Letter by Kumaran and Sharma Regarding “Spatial Transcriptome Analysis of B7-H4 in Head and Neck Squamous Cell Carcinoma: A Novel Therapeutic Target for Anti-immune Checkpoint Inhibitors”

**DOI:** 10.1007/s12105-025-01854-3

**Published:** 2025-10-22

**Authors:** Yuri Noda, Masao Yagi, Koji Tsuta

**Affiliations:** 1 Department of Pathology and Laboratory Medicine, Kansai Medical University Hospital, 2-3-1 Shin-machi, Hirakata, Osaka 573-1191 Japan; 2 Department of Pathology, Kansai Medical University, 2-5-1 Shin-machi, Hirakata, Osaka 573-1010 Japan; 3 Department of Otolaryngology, Head and Neck Surgery, Kansai Medical University Hospital, 2-3-1 Shin-machi, Hirakata, Osaka 5731191 Japan

## Dear Editor

We sincerely thank Kumaran and Sharma for their interest in our recent article, “Spatial Transcriptome Analysis of B7-H4 in Head and Neck Squamous Cell Carcinoma: A Novel Therapeutic Target for Anti-Immune Checkpoint Inhibitors” [1]. We appreciate their constructive remarks and welcome the opportunity to clarify certain aspects of this study. Their letter focused on three issues: (i) the lack of immune-related terms in Gene Ontology (GO) analysis, (ii) the limited number of spatial transcriptomic (ST) cases, and (iii) the absence of a direct correlation with the clinical outcome.

Our primary objective was to determine whether B7-H4 (*VTCN1*) and PD-L1 (*CD274*) exhibit mutually exclusive expression in head and neck squamous cell carcinoma (HNSCC) and to characterize their distribution patterns in the tumor microenvironment. However, this spatial question cannot be answered using bulk transcriptomic datasets lacking tissue architecture. Although databases such as The Cancer Genome Atlas Program (TCGA) provide valuable large-scale information, they do not capture whether protein and mRNA signals overlap within defined epithelial compartments. Conversely, ST integrates mRNA expression with histology, enabling region-specific validation of protein signals. The present study was designed to address this gap.

Big data investigations on B7-H4 have been performed using large-scale datasets. For example, Chang et al. [2] analyzed TCGA HNSCC data and demonstrated that *VTCN1* is among the dysregulated B7/CD28 family genes. Borgmann et al. [3] further reported that B7-H3 and B7-H4 may have prognostic relevance in head and neck cancer. These findings establish B7-H4 as a relevant biomarker in big data cohorts. However, neither TCGA-based analyses nor outcome-driven studies have addressed the spatial concordance of protein and mRNA expression, the central focus of our study.

Regarding sample size and study design, we acknowledge that larger case numbers would strengthen the analysis and have stated this as a limitation [1]. However, our study was not a simple six-case analysis. We performed ST on six patients, each contributing a matched triplet of normal (NOM), squamous intraepithelial neoplasia (SIN), and HNSCC tissues, yielding 18 cores. This within-patient design enabled robust comparisons across normal, premalignant, and malignant compartments, effectively minimizing interpatient heterogeneity. Notably, numerous ST studies on solid tumors have analyzed fewer than 20 cores, supporting the appropriateness of our sample size.

Regarding GO and immune pathway analyses, GO enrichment analysis was conducted on *VTCN1*-positive regions. Immune-related terms were not enriched; instead, terms linked to homeostatic and regulatory processes were identified. This finding should not be interpreted as an absence of immune relevance but suggests that B7-H4 is associated with immune evasion in low-CD8 environments. Accordingly, the biology of immune checkpoint ligands such as B7-H4 may manifest as regulatory alterations that are not readily captured by conventional GO categories.

A key strength of our study is the concordance between B7-H4 and PD-L1 protein domains and their respective transcriptomic domains, confirming mutually exclusive patterns at both levels. This dual validation underscores the robustness of the findings. While prior studies have relied on immunohistochemistry or bulk transcriptomics alone, to our knowledge, no previous study has integrated ST with protein expression in HNSCC. Thus, our study offers a unique perspective on tumor immune heterogeneity in situ.

We acknowledge limitations, including the single-institution cohort, the limited number of *VTCN1*-positive ST cores, and the lack of outcome data. These do not undermine our conclusions but rather define the scope of interpretation. Our findings provide a rationale for further studies in larger multi-institutional cohorts with outcome annotations.

In summary, our study addresses a spatial biology question that cannot be resolved using bulk datasets. Using matched NOM-SIN-HNSCC triplets from six patients (18 cores), we reduced interpatient heterogeneity and confirmed B7-H4/PD-L1 exclusivity at both protein and mRNA levels. Although prior big data studies have considered B7-H4, none have directly demonstrated its spatial distribution. Our study provides novel evidence supporting B7-H4 as a therapeutic target in immune checkpoint-resistant HNSCC.

We appreciate the constructive feedback offered by Kumaran and Sharma, and hope that this clarification allows readers to better understand the aims, strengths, and limitations of our study.

Sincerely,

Yuri Noda, Masao Yagi, Koji Tsuta (on behalf of all authors).

## Data Availability

No datasets were generated or analysed during the current study.
